# Anaerobic Capacity in Running: The Effect of Computational Method

**DOI:** 10.3389/fphys.2021.708172

**Published:** 2021-08-04

**Authors:** Erik P. Andersson, Glenn Björklund, Kerry McGawley

**Affiliations:** ^1^Swedish Winter Sports Research Centre, Department of Health Sciences, Mid Sweden University, Östersund, Sweden; ^2^School of Sport Sciences, Faculty of Health Sciences, UiT The Arctic University of Norway, Tromsø, Norway

**Keywords:** gross energy cost, MAOD, maximal accumulated oxygen deficit, metabolic demand, running economy, time trial, supramaximal exercise

## Abstract

**Introduction:**

To date, no study has compared anaerobic capacity (AnC) estimates computed with the maximal accumulated oxygen deficit (MAOD) method and the gross energy cost (GEC) method applied to treadmill running exercise.

**Purpose:**

Four different models for estimating anaerobic energy supply during treadmill running exercise were compared.

**Methods:**

Fifteen endurance-trained recreational athletes performed, after a 10-min warm-up, five 4-min stages at ∼55–80% of peak oxygen uptake, and a 4-min time trial (TT). Two linear speed-metabolic rate (MR) regression models were used to estimate the instantaneous required MR during the TT (MR_*TT_req*_), either including (5+Y_*LIN*_) or excluding (5-Y_*LIN*_) a measured Y-intercept. Also, the average GEC (GEC_*AVG*_) based on all five submaximal stages, or the GEC based on the last submaximal stage (GEC_*LAST*_), were used as models to estimate the instantaneous MR_*TT_req*_. The AnC was computed as the difference between the MR_*TT_req*_ and the aerobic MR integrated over time.

**Results:**

The GEC remained constant at ∼4.39 ± 0.29 J⋅kg^–1^⋅m^–1^ across the five submaximal stages and the TT was performed at a speed of 4.7 ± 0.4 m⋅s^–1^. Compared with the 5-Y_*LIN*_, GEC_*AVG*_, and GEC_*LAST*_ models, the 5+Y_*LIN*_ model generated a MR_*TT_req*_ that was ∼3.9% lower, with corresponding anaerobic capacities from the four models of 0.72 ± 0.20, 0.74 ± 0.16, 0.74 ± 0.15, and 0.54 ± 0.14 kJ⋅kg^–1^, respectively (*F*_1.07,42_ = 13.9, *P* = 0.002). The GEC values associated with the TT were 4.22 ± 0.27 and 4.37 ± 0.30 J⋅kg^–1^⋅m^–1^ for 5+Y_*LIN*_ and 5-Y_*LIN*_, respectively (calculated from the regression equation), and 4.39 ± 0.28 and 4.38 ± 0.27 J⋅kg^–1^⋅m^–1^ for GEC_*AVG*_ and GEC_*LAST*_, respectively (*F*_1.08,42_ = 14.6, *P* < 0.001). The absolute typical errors in AnC ranged between 0.03 and 0.16 kJ⋅kg^–1^ for the six pair-wise comparisons and the overall standard error of measurement (SEM) was 0.16 kJ⋅kg^–1^.

**Conclusion:**

These findings demonstrate a generally high disagreement in estimated anaerobic capacities between models and show that the inclusion of a measured Y-intercept in the linear regression (i.e., 5+Y_*LIN*_) is likely to underestimate the MR_*TT_req*_ and the GEC associated with the TT, and hence the AnC during maximal 4-min treadmill running.

## Introduction

From a physiological perspective, speed of locomotion in endurance sports such as running and cycling is primarily related to the maximal aerobic metabolic rate (MR) (i.e., maximum oxygen uptake, $\dot{\text{V}}{\text{O}}_{2max}$), its fractional utilization, and the energy cost of movement. Although aerobic energy provision is the primary source of energy supply in endurance sports, anaerobic energy provision is also involved (Joyner and Coyle, 2008). Due to the limited capacity of the anaerobic energy supply, the relative anaerobic contribution to exercise decreases with duration (Gastin, 2001). For example, in 800 and 1500-m track running (lasting ∼2.1 and ∼4.4 min, respectively), the relative contribution of anaerobic metabolism decreases from ∼40 to ∼14% in trained runners (Duffield et al., 2005a,b). In addition, many endurance races are often performed head-to-head, making anaerobic energy provision crucial for the success of breakaways and final end-spurts.

Although aerobic energy provision can be quantified during exercise by using measures of oxygen consumption and carbon dioxide production, the quantification of anaerobic energy provision is more complicated. Several different methods of quantifying anaerobic capacity (AnC) have been used (Noordhof et al., 2013), but the gold standard procedure is probably the direct method first described by Bangsbo et al. (1990) for single-leg knee extension exercise. However, this sophisticated invasive method is not applicable for traditional whole-body endurance modalities such as running or cycling, where many large muscle groups are involved in locomotion (Noordhof et al., 2010). Therefore, indirect estimates of anaerobic energy production are commonly used, such as the maximal accumulated oxygen deficit (MAOD) method (Medbø et al., 1988) or the gross efficiency (GE) method (Serresse et al., 1988; Noordhof et al., 2011; Andersson and McGawley, 2018). The MAOD method identifies a supramaximal total average oxygen uptake ($\dot{\text{V}}{\text{O}}_{2}$) requirement calculated from a linear relationship between submaximal $\dot{\text{V}}{\text{O}}_{2}$ and speed (or power output). Due to the effect of increasing submaximal exercise intensity on substrate utilization (Shaw et al., 2014) and the different energy equivalents for fat and carbohydrate oxidation (Weir, 1949), a speed (or power output) vs. MR relationship should be more appropriate (Andersson and McGawley, 2018). By contrast, for the GE method, the supramaximal total metabolic requirement can be calculated by dividing the supramaximal power output by GE determined from a single submaximal exercise bout. For both methods, the anaerobic MR during supramaximal exercise can be calculated by subtracting the instantaneous aerobic MR from the total required instantaneous MR, with the AnC calculated as the anaerobic MR integrated over time (Andersson and McGawley, 2018; Andersson et al., 2020).

The main limitations of the linear regression method are related to the range of exercise intensities included in the submaximal linear relationship, the number, and duration of the submaximal stages, and whether a continuous or discontinuous submaximal protocol is used (Noordhof et al., 2010). In addition, the effect of including a baseline value of MR in the linear regression model is equivocal and might differ for exercise modalities (Bangsbo, 1992, 1996; Noordhof et al., 2011; Andersson and McGawley, 2018; Andersson et al., 2020). For instance, Andersson et al. (2020) showed for diagonal-stride treadmill roller-skiing that the inclusion of a resting baseline value of MR (i.e., the Y-intercept value) was likely to result in an underestimated supramaximal metabolic requirement and AnC compared to no inclusion of a baseline Y-intercept value. However, Noordhof et al. (2011) showed no such difference when including a fixed Y-intercept value for cycle ergometry exercise.

Due to the difficulties in determining external work during running, the GE method can be considered inappropriate for determining AnC for running exercise. Therefore, most previous studies that have estimated AnC during treadmill running have used the MAOD approach (Medbø et al., 1988; Ramsbottom et al., 1994; Craig and Morgan, 1998; Spencer and Gastin, 2001; Hill et al., 2002) while the GE approach has been more commonly used during cycle ergometry exercise to estimate anaerobic work capacity and/or anaerobically attributable power output (Serresse et al., 1988; Foster et al., 2003; de Koning et al., 2005; Hettinga et al., 2006). An alternative method that can be used to estimate the AnC for treadmill roller-skiing or running is the gross energy cost (GEC, J⋅kg^–1^⋅m^–1^) method, which is conceptually similar to the GE method and has recently been employed for estimating AnC during diagonal-stride treadmill roller-skiing (Andersson and McGawley, 2018). In this study, both the GE and GEC methods resulted in identical values of AnC and are, thus, conceptually similar when applied to treadmill exercise in a laboratory where air drag is negligible. Therefore, the GEC concept may potentially be used as an alternative to the GE approach when estimating AnC during supramaximal running on a treadmill. A potential limitation of the GEC (or GE) method is the assumption that GEC (or GE) is speed (or power output) independent (Batliner et al., 2018; Andersson et al., 2020). Based on the linear regression between speed (or power output) and MR, as used in the modified MAOD method, GEC (or GE) is only constant when the Y-intercept is zero. For relationships with a positive Y-intercept, GEC will always decrease (or GE will increase) with increasing exercise intensity due to the diminishing effect of the Y-intercept value (Ettema and Lorås, 2009; Batliner et al., 2018). However, when a wide range of exercise intensities are used and combined with a resting baseline value in the linear MAOD regression, a positive Y-intercept would be expected (Noordhof et al., 2011), which in theory would explain the disagreement between the MAOD and GE methods (Andersson et al., 2020).

At present, a limited number of studies have compared different AnC estimates generated by the MAOD and GE methods (Noordhof et al., 2011; Andersson and McGawley, 2018; Andersson et al., 2020). The overall conclusion of these studies is that the different methods should not be used interchangeably, due to the relatively high within-participant disagreement. However, there is sparse information explaining this disagreement or the typical errors associated with the different AnC estimates generated by the MAOD and GEC/GE concepts. To date, there is, to our knowledge, only one study that has provided any methodological interpretation of the within-participant disagreement between the GE and MAOD methods, which was related to the individual variation in the Y-intercept values for the MAOD regression (Andersson et al., 2020). Given this sparsity of methodological inquiry, the current study aimed to compare estimates of anaerobic capacities generated during a 4-min running performance time trial (TT) using four different models: the 5 × 4-min MAOD method with the inclusion of a baseline Y-intercept value (5+Y_*LIN*_) and without the inclusion of a baseline Y-intercept value (5-Y_*LIN*_), and two GEC methods using the average GEC based on the 5 × 4-min submaximal exercise intensities (GEC_*AVG*_) and using the last exercise intensity only (GEC_*LAST*_).

## Materials and Methods

### Participants

Fifteen endurance-trained recreational athletes (seven women and eight men; mean ± standard deviation (SD): age 31.3 ± 6.7 years, body mass 70.8 ± 10.5 kg) volunteered to participate in this study, which was preapproved by the Regional Ethical Review Board of Umeå University, Umeå, Sweden. All participants were fully informed about the study before providing written consent to participate. The participants were recruited from local running, cross-country skiing, triathlon, and multi-sport clubs. Immediately prior to the start of the study participants were carrying out high-intensity interval training weekly or bi-weekly and they competed in a mixture of running, cross-country skiing, and/or multi-sport competitions.

### Study Overview

The participants completed a continuous submaximal treadmill running protocol consisting of 5 × 4-min stages, ranging from ∼9.7–13.2 km⋅h^–1^ (∼55–80% of estimated peak $\dot{\text{V}}{\text{O}}_{2}$[$\dot{\text{V}}{\text{O}}_{2peak}$]) and with step increments of ∼1 km⋅h^–1^ per stage, and a 4-min TT. Both the submaximal protocol and the TT were performed on a motorized treadmill set at a 1% incline. Participants were instructed to self-select the pace of the 4-min TT by moving to the front or rear of the treadmill, which was equipped with a bespoke speed-controlling laser system that allows the athlete to freely adjust the speed by moving forward or backward on the treadmill, which makes TT tests possible. They were also instructed to cover as much distance as possible in the fixed time and received only time-related feedback during the 4 min. The 4-min TT was performed at least 10 min after the submaximal test, and following a 10-min re-warm-up. The submaximal speeds were based on previous 5- and/or 10-km running race performances, or previously measured $\dot{\text{V}}{\text{O}}_{2peak}$ or $\dot{\text{V}}{\text{O}}_{2max}$ in running or diagonal roller-skiing, and assuming a GEC of running at 4.3 J⋅kg^–1^⋅m^–1^ as based on previously unpublished test results.

### Equipment and Measurements

All tests were performed on a treadmill (Rodby Innovation AB, Vänge, Sweden) whereby distance completed during the TT was automatically logged at a rate of 2.46 Hz and linearly interpolated to second-by-second data. Participants were secured with a safety harness suspended from the ceiling and connected to an emergency brake when exercising on the treadmill, which immediately stopped in the case of a fall. Respiratory measurements were performed using an AMIS 2001, model C (Innovision AS, Odense, Denmark). The gas analyzers were calibrated with a known reference gas containing 16.0% O_2_ and 4.5% CO_2_ (Air Liquide, Kungsängen, Sweden) and ambient air. The flow meter was calibrated before the start of each test with a 3-L syringe at low, medium, and high flow rates (Hans Rudolph, Kansas City, MO, United States). Heart rate was monitored using a chest strap and wristwatch (V800 or RS800CX, Polar Electro Oy, Kempele, Finland). Blood lactate concentration was determined using a Biosen C_Line or S_Line (EKF diagnostics, Magdeburg, Germany) calibrated with a known standard solution of 12 mmol⋅L^–1^.

### Testing Procedures

#### The Submaximal Test

The participants reported to the laboratory rested, in a fed state, and having abstained from alcohol and intense training for at least 24 h before testing, and from caffeine on the day of testing. Participants’ body mass was measured using an electronic scale (Seca 764, Hamburg, Germany) in the same clothing as worn during the tests. After ∼5 min of seated rest a 2-min baseline $\dot{\text{V}}{\text{O}}_{2}$ measurement was collected with the participant standing still on the treadmill, after which the exercise test began. The submaximal protocol was conducted as described previously (Watkins et al., 2017), commencing with a 10-min warm-up followed by a series of 4-min incremental stages, which increased by ∼1 km⋅h^–1^ every 4 min (with incline fixed at 1%). At the end of each 4-min stage, a fingertip blood sample (for blood lactate assessment) and a rating of perceived exertion [RPE: 6–20 point scale (Borg, 1982)] were collected. The test was terminated after at least five stages when participants had reached an RPE of 15–17. Heart rate was recorded throughout the test and was averaged over the last 30 s of each stage, while respiratory variables were averaged between 2 min 50 s and 3 min 50 s of each stage. If more than five submaximal stages were completed, the five stages that were closest to an intensity range of 55–80% $\dot{\text{V}}{\text{O}}_{2peak}$ (calculated from the 4-min TT) were used for further analyses.

#### The 4-Min TT

After at least 10 min of rest following the submaximal test, participants completed a 10-min re-warm-up and a 4-min TT as previously described (Watkins et al., 2017). Briefly, the warm-up consisted of 5 min of low-intensity running, 3 × 30-s intervals separated by 30 s of low-intensity running, then 2 min of low-intensity running. After a 3-min passive break, the participants commenced the TT at a starting speed equivalent to the speed of the final submaximal stage minus 2 km⋅h^–1^, again at an incline of 1%. The treadmill speed was freely adjusted by the participant moving to the front of the belt to accelerate (at a rate of 0.50 km⋅h^–1^⋅s^–1^), to the rear to decelerate (at a rate of 0.40 km⋅h^–1^⋅s^–1^), or staying in the middle of the belt to maintain a constant speed. The participant was instructed to perform a maximal-paced effort to cover as much distance as possible in the 4 min. Elapsed time was visible on a screen and standardized encouragement was provided, but no further feedback to the participant was available. Respiratory and heart rate data were collected continuously during the TT and the highest 30-s moving average was used to calculate $\dot{\text{V}}$O_2peak_ and peak ventilation rate, while peak heart rate was obtained as the highest 5-s average value. Peak respiratory exchange ratio (RER) was taken over the same period as the $\dot{\text{V}}{\text{O}}_{2peak}$. Fingertip blood samples were taken (for blood lactate assessment) at 1, 2, 3, and 4 min after the 4-min TT, with the highest value reported as the peak.

### Calculations

#### Submaximal Running

Energy expenditure was calculated from $\dot{\text{V}}$O_2_ and RER ($\dot{\text{V}}$CO_2_⋅$\dot{\text{V}}$O_2_^–1^) according to the equation introduced by Weir (1949) and subsequently converted into a MR. MR was based on the average $\dot{\text{V}}$O_2_ in mL⋅kg^–1^⋅min^–1^ and RER values (≤1.00) during the final minute of each stage of the submaximal exercise protocol.

(1) $$Metabolicrate\left[W\cdot kg^{-1}\right]=\frac{4.184\dot{V}O_{2}\left(1.1RER+3.9\right)}{60}$$

GEC was calculated as:

(2) $$GEC\left[J\cdot kg^{-1}\cdot m^{-1}\right]=\frac{Metabolicrate\left[W\cdot kg^{-1}\right]}{Speed\left[m\cdot s^{-1}\right]}$$

Net energy cost was calculated as:

(3) $$Netenergycost\left[J\cdot kg^{-1}\cdot m^{-1}\right]=\frac{Metabolicrate-MR_{BL}\left[W\cdot kg^{-1}\right]}{Speed\left[m\cdot s^{-1}\right]}$$

where MR_*BL*_ is the baseline MR calculated from 1-min baseline $\dot{\text{V}}{\text{O}}_{2}$ and RER measurements when the participant stood still on the treadmill (prior to the warm-up). Delta energy cost (J⋅kg^–1^⋅m^–1^) was calculated by dividing the increase in MR (W⋅kg^–1^) by the increase in speed (m⋅s^–1^) based on the linear regression between MR and speed over the five submaximal exercise intensities, which is identical to the value of the slope of the regression equation. Neither net energy cost nor delta energy cost was used for estimating the AnC. The $\dot{\text{V}}{\text{O}}_{2peak}$ during the TT was converted to a peak aerobic MR by using Eq. 1 and assuming 100% carbohydrate utilization (i.e., using an RER of 1.00).

#### Estimating AnC

A linear relationship between treadmill speed and MR (W⋅kg^–1^) during the final min of each of the 5 × 4-min submaximal stages was derived for each participant with the baseline MR as a Y-intercept (i.e., the MR at zero speed) included in (5+Y_*LIN*_) or excluded from (5-Y_*LIN*_) the model. In the latter case, the Y-intercept was based on all data points in the regression (i.e., not forced). The two regression equations were used to estimate the required instantaneous MR during the 4-min TT (MR_*TT_req*_) at each 1-s time-point.

The submaximal GEC calculated as an average of all the submaximal stages (GEC_*AVG*_) or from the last submaximal stage only (GEC_*LAST*_) were also used to estimate the MR_*TT_req*_ at each 1-s time-point of the TT. Here, the MR_*TT_req*_ was calculated by multiplying instantaneous TT speed (in m⋅s^–1^) with a fixed GEC value (i.e., GEC_*AVG*_ or GEC_*LAST*_).

For all four methods (i.e., 5+Y_*LIN*,_ 5-Y_*LIN*,_ GEC_*AVG*,_ and GEC_*LAST*_), the instantaneous anaerobic MR (MR_*an*_) at each 1-s time-point (*t*) of the TT could then be expressed as:

(4) $$MR_{an,t}\left[W\cdot kg^{-1}\right]MR_{TT\_req,t}-MR_{ae,t}$$

where MR_*ae*_ is the aerobic MR calculated according to Eq. 1.

For all four methods, the AnC (J⋅kg^–1^) was calculated by integrating MR_*an*_ over the 4-min TT. The anaerobic energy production was, in addition, converted to an accumulated oxygen deficit by multiplying the AnC with a constant of 0.047801 (mL oxygen equivalent per joule) according to Weir (1949) and assuming 100% carbohydrate utilization during the supramaximal TT. In the Supplementary Tables, AnC estimates based on two polynomial models (5+Y_*POL*_ and 5-Y_*POL*_) are also presented. The MR_*TT_req*_ for these two additional models was determined similarly as for the two linear models (i.e., 5+Y_*LIN*_ and 5-Y_*LIN*_), but using a second-degree polynomial regression equation rather than a linear regression equation. In addition to the 5+Y_*POL*_ and 5-Y_*POL*_ models, data for three alternative linear models are also presented in the Supplementary Tables, based on: (1) the four highest submaximal stages (4-Y_*LIN*_); (2) the three highest submaximal stages (3-Y_*LIN*_); and (3) the two highest submaximal stages (2-Y_*LIN*_). The results generated by these five alternative models were compared with the 5-Y_*LIN*_ and GEC_*LAST*_ models (Supplementary Tables 1, 2).

#### Comparing the Measured GEC With GEC Derived From the Two Regression Equations (GEC_*REG*_)

The GEC based on each of the two regression equations (GEC_*REG*_, based on the 5+Y_*LIN*_ and 5-Y_*LIN*_ models) was calculated for the five submaximal stages as MR, calculated from the regression equation, divided by speed. This enabled a comparison of the measured GEC during the five submaximal stages with the GEC_*REG*_. To compare the average supramaximal GEC_*REG*_ during the TT with the GEC_*AVG*_ and GEC_*LAST*_ values, the following calculations were performed: firstly, the estimated instantaneous GEC at each 1-s time-point (*t*) of the 4-min TT was calculated for 5+Y_*LIN*_ and 5-Y_*LIN*_ as MR_*TT_req*_ (derived from the linear regression equation) divided by speed; secondly, the estimated instantaneous GEC during the TT was expressed as an average value for each of the two respective models. The same methods were used for the additional models that are presented in the Supplementary Tables.

### Statistics

All statistical tests were processed using Office Excel 2016 (Microsoft Corporation, Redmond, WA, United States) and the Statistical Package for the Social Sciences (SPSS 25, IBM Corp., Armonk, NY, United States). The level of statistical significance was set at α ≤ 0.05. Data were checked for normality by visual inspection of Q-Q plots and histograms together with the Shapiro–Wilks analysis and are presented as mean ± SD, except in the case of RPE and heart rate, where data are presented as median and interquartile range (IQR). In addition, the different AnC estimates were presented as mean and 95% confidence intervals. The linear relationships between submaximal speed and MR for the 5+Y_*LIN*_ and 5-Y_*LIN*_ models were assessed using linear regression analyses. One-way repeated measures ANOVA tests were used to compare GEC and net energy cost between the five submaximal stages as well as the GEC, required MR, and AnC associated with the TT. A paired *t*-test was used to analyze the linear regression coefficients for the 5+Y_*LIN*_ and 5-Y_*LIN*_ models. The precision of the two linear regression equations was assessed with the standard error of the estimate (SEE). The root mean square error was used to evaluate the relative discrepancy between GEC calculated from the two regression equations and GEC measured during the five submaximal running stages expressed as a percentage error. For the ANOVA tests, the assumption of sphericity was assessed using Mauchly’s test. For violated sphericity, a Greenhouse–Geisser correction of the degrees of freedom was used (epsilon ≤ 0.75). Bonferroni α corrections were applied to all ANOVA tests and eta squared effect sizes (η^2^) were also reported.

The mean difference ± 95% limits of agreement for the comparison of the four AnC estimates were evaluated using Bland–Altman calculations (Bland and Altman, 1999). The mean difference was tested with a paired-sample *t*-test and the standardized mean difference [Hedges’ *g*_*av*_ effect size (*Hg*_*av*_)] was computed according to the equations presented by Lakens (2013). In addition, the methodological error was evaluated via the overall standard error of measurement (SEM) calculated based on the intraclass correlation coefficient. The intraclass correlation coefficient was calculated as the between-subjects mean square value minus the within-subjects mean square value and divided by the between-subjects mean square value from the repeated-measures ANOVA. The absolute typical error was calculated for all the separate pair-wise comparisons.

## Results

The submaximal speeds, physiological responses, and two various concepts of energy cost (i.e., GEC and net energy cost) at the five submaximal stages are shown in Table 1. The GEC remained unchanged (∼4.39 J⋅kg^–1^⋅m^–1^) between all submaximal stages (*F*_1.48,56_ = 0.84, *P* = 0.413, and η^2^ = 0.004) whereas the net energy cost increased by ∼4.4% from the first to the last submaximal stage (*F*_1.55,56_ = 11.0, *P* < 0.001, and η^2^ = 0.048).

**TABLE 1:** Mean ± standard deviation (SD) speeds, heart rates, cardiorespiratory variables, blood lactate concentrations, and energy costs associated with the five submaximal stages (SUB_1–5_) of treadmill running at a 1% incline, as well as the stand-up resting baseline (BL_*REST*_) data.

	BL_*REST*_	SUB_1_	SUB_2_	SUB_3_	SUB_4_	SUB_5_
Speed (m⋅s^–1^)	0	2.60 ± 0.24	2.87 ± 0.24	3.13 ± 0.24	3.40 ± 0.24	3.66 ± 0.25
Heart rate (% of max)	42 ± 4	72 ± 3	76 ± 3	81 ± 3	85 ± 4	90 ± 4
MR_*AE*_ (W⋅kg^–1^)	1.8 ± 0.2	11.5 ± 1.1	12.6 ± 1.2	13.7 ± 1.3	14.9 ± 1.3	16.1 ± 1.4
MR_*AE*_ (% of MR_*AE_peak*_)	9 ± 2	57 ± 3	62 ± 4	68 ± 3	73 ± 4	79 ± 4
Ventilation rate (L⋅min^–1^)	13.7 ± 2.7	60.6 ± 10.8	66.3 ± 10.8	73.2 ± 11.6	81.5 ± 13.0	90.6 ± 14.1
$\dot{\text{V}}$_*E*_⋅$\dot{\text{V}}\text{CO}$_2_^–1^	42.1 ± 6.3	28.3 ± 2.7	28.3 ± 2.9	28.3 ± 2.6	28.6 ± 2.5	29.0 ± 2.8
$\dot{\text{V}}$_*E*_⋅$\dot{\text{V}}\text{O}$_2_^–1^	37.4 ± 7.0	25.6 ± 2.8	25.6 ± 2.9	26.0 ± 2.3	26.7 ± 2.2	27.6 ± 2.3
RER ($\dot{\text{V}}$CO_2_⋅$\dot{\text{V}}$O_2_^–1^)	0.88 ± 0.10	0.90 ± 0.05	0.91 ± 0.05	0.92 ± 0.05	0.93 ± 0.05	0.95 ± 0.04
La^–^ (mmol⋅L^–1^)	–	1.1 ± 0.2	1.2 ± 0.3	1.3 ± 0.2	1.7 ± 0.5	2.2 ± 0.7
Gross energy cost (J⋅kg^–1^⋅m^–1^)	–	4.42 ± 0.31	4.39 ± 0.31	4.37 ± 0.27	4.38 ± 0.26	4.38 ± 0.27
Net energy cost (J⋅kg^–1^⋅m^–1^)	–	3.73 ± 0.28	3.76 ± 0.29	3.80 ± 0.26	3.85 ± 0.25	3.89 ± 0.27

*MR_*AE*_, aerobic metabolic rate; MR_*AE_peak*_, peak aerobic metabolic rate; $\dot{\text{V}}$_*E*_⋅$\dot{\text{V}}\text{CO}$_2_^–1^, ventilatory equivalent for carbon dioxide; $\dot{\text{V}}$_*E*_⋅$\dot{\text{V}}\text{O}$_2_^–1^, ventilatory equivalent for oxygen; RER, respiratory exchange ratio; La^–^, blood lactate concentration.*

**FIGURE 1:**
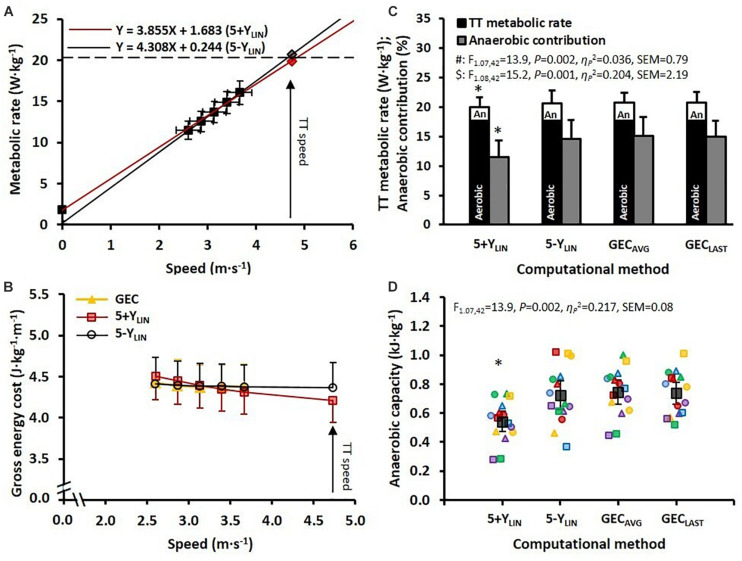
**(A)** The two regression models for mean ± SD speed and metabolic rate (MR) (relative to body mass) during 5 × 4-min stages of continuous submaximal running, together with the estimated total metabolic requirements (diamonds) at the average speed attained during the 4-min time trial (TT). Red line: 5+Y_*LIN*_; black line: 5-Y_*LIN*_. **(B)** Mean ± SD gross energy cost (GEC) for the 5 × 4-min stages of submaximal running (yellow triangles) and GEC calculated from the two regression equations (red squares: 5+Y_*LIN*_; open circles: 5-Y_*LIN*_) for the submaximal stages and the TT. **(C)** Mean ± standard deviation (SD) total MR during the TT with the aerobic and anaerobic (An) contributions and the relative anaerobic contribution (expressed as a percentage) for the two linear regression models (i.e., 5+Y_*LIN*_ and 5-Y_*LIN*_) and when using the average GEC based on the five submaximal stages (GEC_*AVG*_) and the last submaximal stage (GEC_*LAST*_). **(D)** Anaerobic capacity (AnC) expressed as mean and 95% confidence intervals (dark filled squares and bars), together with individual data (colored symbols). *Significantly lower than 5-Y_*LIN*_, GEC_*AVG*_, and GEC_*LAST*_, all *P* < 0.001.

The mean ± SD speed and MR during the five submaximal stages and the TT, together with the regression lines (based on the mean values), are displayed in Figure 1A for the 5+Y_*LIN*_ and 5-Y_*LIN*_ models. The mean ± SD values of GEC and the GEC calculated from the 5+Y_*LIN*_ and 5-Y_*LIN*_ models are displayed in Figure 1B. The 4-min TT was completed at an average speed of 4.7 ± 0.4 m⋅s^–1^ (17.0 ± 1.5 km⋅h^–1^). The $\dot{\text{V}}{\text{O}}_{2peak}$ was 58 ± 6 mL⋅kg^–1^⋅min^–1^ (4.2 ± 0.9 L⋅min^–1^), with an RER of 1.14 ± 0.05. The peak ventilation rate, peak heart rate, and peak blood lactate concentration were 148 ± 26 L⋅min^–1^, 183 (IQR = 177–188) beats⋅min^–1^, and 11.0 ± 2.1 mmol⋅L^–1^, respectively. The RPE measured immediately after the TT was 19 (IQR = 18–19).

The estimated total MRs during the TT (including the aerobic and anaerobic contributions) and the AnC values are shown in Figures 1C,D, respectively, for the four different computational methods. In Figure 1D it can be seen that the estimated AnC was considerably lower for the 5+Y_*LIN*_ model compared to the three other models (i.e., 5-Y_*LIN*_, GEC_*AVG*_, and GEC_*LAST*_).

The data presented in Table 2 show that the SEE was approximately twice as large for the 5+Y_*LIN*_ vs. the 5-Y_*LIN*_ regression model. Also, the Y-intercept value was considerably higher for the 5+Y_*LIN*_ model, which resulted in a regression slope (i.e., a delta energy cost) that was ∼10% lower for the 5+Y_*LIN*_ model than for the 5-Y_*LIN*_ model. The root mean square error for the relative difference between GEC calculated from the speed-MR regression and the GEC measured during the five submaximal stages was 1.8 ± 0.8% for the 5+Y_*LIN*_ and 0.8 ± 0.3% for the 5-Y_*LIN*_ model (*P* < 0.001, *Hg*_*av*_ = 1.6). The average GEC during the TT was ∼3.7% lower for the 5+Y_*LIN*_ compared to the other three models, which resulted in an ∼3.9% lower required MR during the TT for the 5-Y_*LIN*_ model (see Table 2). This explains the ∼26% lower estimated AnC for the 5+Y_*LIN*_ model (illustrated in Figure 1D).

**TABLE 2:** Mean ± SD slope, Y-intercept, coefficient of determination (*r*^2^), standard error of estimate (SEE) for the two linear models, and gross energy cost (GEC), metabolic requirement, and accumulated oxygen deficit (ΣO_2_ deficit) during the 4-min time trial (TT) for the four different models of estimating the anaerobic capacity (AnC).

Method of calculation								
	5+Y_*LIN*_	5-Y_*LIN*_	GEC_*AVG*_	GEC_*LAST*_	Test statistic	*P*-value	ES	SEM
Slope (W⋅kg^–1^ per m⋅s^–1^)	3.85 ± 0.26	4.32 ± 0.56	–	–	–	*P* = 0.002	*Hg*_*av*_ = −1.0	–
Y-intercept (W⋅kg^–1^)	1.69 ± 0.25	0.18 ± 1.71	–	–	–	*P* = 0.003	*Hg*_*av*_ = 1.2	–
*r* ^2^	0.997 ± 0.002	0.996 ± 0.003	–	–	–	*P* = 0.156	*Hg*_*av*_ = 0.5	–
SEE (W⋅kg^–1^)	0.28 ± 0.13	0.13 ± 0.05	–	–	–	*P* < 0.001	*Hg*_*av*_ = 1.5	–
GEC_*TT_avg*_ (J⋅kg^–1^⋅m^–1^)	4.22 ± 0.27*	4.37 ± 0.30	4.39 ± 0.28	4.38 ± 0.27	*F*_1.08,42_ = 14.6	*P* < 0.001	η^2^ = 0.063	0.16
MR_*TT_req*_ (% of MR_*ae_peak*_)	98 ± 3*	102 ± 5	102 ± 4	102 ± 4	*F*_1.11,42_ = 15.2	*P* < 0.001	η^2^ = 0.160	4.16
ΣO_2_ deficit (mL⋅kg^–1^)	26 ± 7*	35 ± 9	36 ± 8	35 ± 7	*F*_1.07,42_ = 13.9	*P* = 0.002	η^2^ = 0.217	3.92

5+Y_*LIN*_ and 5-Y_*LIN*_, the 5 × 4-min linear methods with the baseline metabolic rate as a Y-intercept either included (5+Y) or excluded (5-Y); GEC_*AVG*_, the gross energy cost procedure based on the average value of five submaximal stages; GEC_*LAST*_, the gross energy cost procedure based on the last submaximal stage; ES, effect size; SEM, standard error of measurement; GEC_*TT*_*avg*_, average GEC during the TT; MR_*TT*_*req*_, required metabolic rate during the TT; MR_*ae_peak*_, peak aerobic metabolic rate during the TT. *F*-values, *P*-values, and eta squared effect size (η^2^) were obtained by a one-way ANOVA. *Significantly lower than 5-Y_*LIN*_, GEC_*AVG*_, and GEC_*LAST*_, all *P* < 0.001.

**FIGURE 2:**
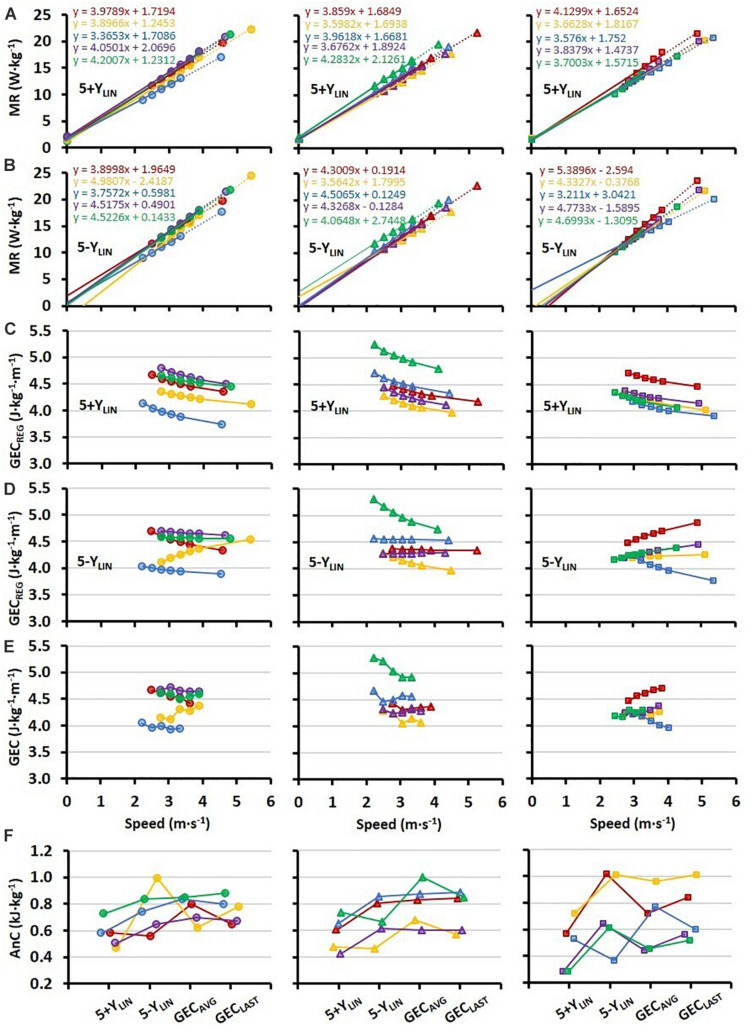
Individual regressions (*N* = 15, 5 in each of the three horizontal panels) for: **(A)** submaximal MR plotted against treadmill running speed (at a 1% incline) based on the five 4-min submaximal stages and extrapolation up to the average 4-min TT speed including a Y-intercept value (5+Y_*LIN*_), and **(B)** when excluding the Y-intercept value (5-Y_*LIN*_); **(C,D)** GEC calculated from the two regression equations (GEC_*REG*_) for the submaximal stages and the TT, with panel **(C)** for the 5+Y_*LIN*_ regression and panel **(D)** for the 5-Y_*LIN*_ regression; **(E)** directly measured individual values of GEC for the submaximal stages; **(F)** individual values of AnC calculated with the four different models, where the 5+Y_*LIN*_ and 5-Y_*LIN*_ are the two linear models and the GEC_*AVG*_ being based on the average value of all five submaximal stages and with GEC_*LAST*_ being based on the GEC value from the last submaximal stage.

All individual regression lines between speed and MR are shown in Figures 2A,B for the 5+Y_*LIN*_ and 5-Y_*LIN*_ models, respectively. The GEC calculated from the two regression equations (GEC_*REG*_) for the five submaximal speeds and the TT speed is presented in Figures 2C,D for the 5+Y_*LIN*_ and 5-Y_*LIN*_ models, respectively, whereas the directly measured GEC is presented in Figure 2E. It can be seen that the inclusion of a Y-intercept value in the linear regression (i.e., the 5+Y_*LIN*_ model) resulted in a decline in GEC with increasing speed for all participants, which deviates from the somewhat mixed speed-GEC patterns presented in Figures 2D,E. The within-participant disagreement in estimated AnC for the four different models is illustrated in Figure 2F.

**FIGURE 3:**
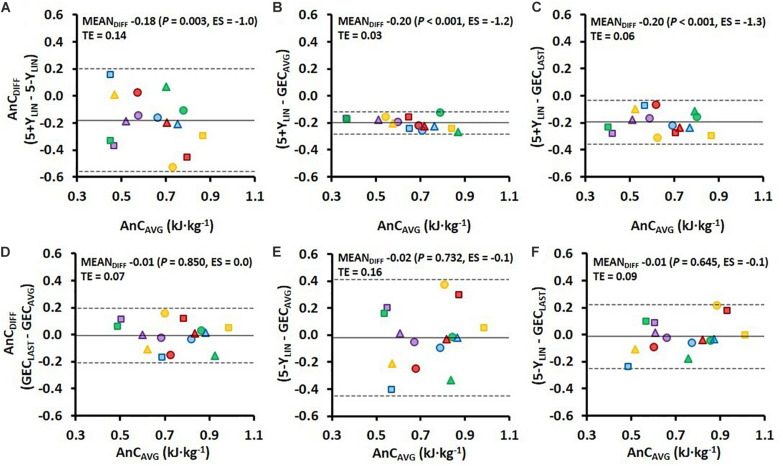
Bland–Altman plots representing the mean difference (MEAN_*DIFF*_) in the AnC ± 95% limits of agreement (i.e., 1.96 SD) associated with the 4-min running TT for the four various models. AnC_*DIFF*_, the difference in AnC; AnC_*AVG*_, the average AnC for the two compared models; TE, typical error; ES, Hedges’s *g*_*av*_ effect size (*Hg*_*av*_), 5+Y_*LIN*_ and 5-Y_*LIN*_, the two linear models; GEC_*AVG*_, the GEC method based on the average of five submaximal stages; and GEC_*LAST*_, the GEC method based on the last submaximal stage.

Comparisons of the AnC estimates from the 4-min TT using the four different models are presented in Figure 3. As shown in Figures 3A–C, the 5+Y_*LIN*_ model generated anaerobic capacities that were ∼19 kJ⋅kg^–1^ lower than the 5-Y_*LIN*_, GEC_*AVG*_, and GEC_*LAST*_ models, whereas the mean difference between the 5-Y_*LIN*_, GEC_*AVG*_, and GEC_*LAST*_ models was approximately zero (Figures 3D–F). The typical errors for the respective comparisons were generally high, with exception of the 5+Y_*LIN*_ vs. GEC_*AVG*_ model (see Figure 3).

**FIGURE 4:**
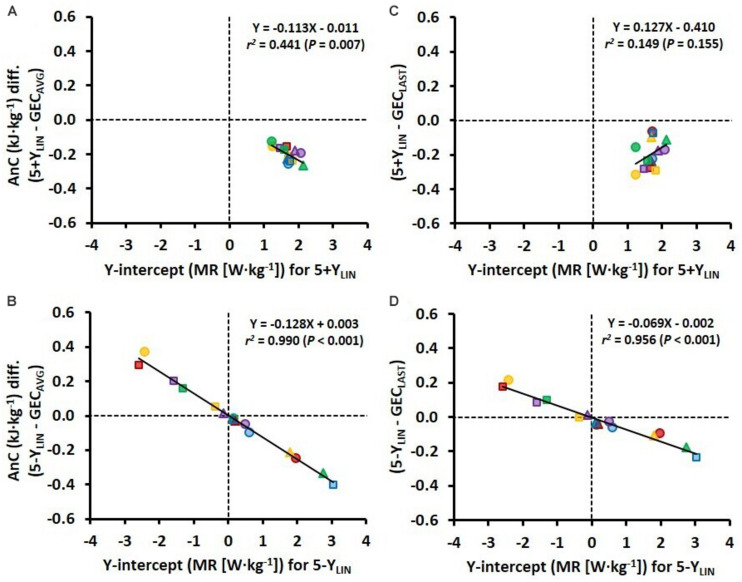
Scatter plots between the Y-intercept values for the 5 × 4-min linear models with the baseline MR as a Y-intercept either included (5+Y_*LIN*_) or excluded (5-Y_*LIN*_) in the model (*x*-axis) and the difference in AnC (AnC diff.) vs. the GEC method based on the average of five submaximal stages (GEC_*AVG*_) (*y*-axis) **(A,B)** and vs. the GEC method based on the last submaximal stage (GEC_*LAST*_) (*y*-axis) **(C,D)**.

The variation in Y-intercept values for the 5-Y_*LIN*_ model was highly related to the variation in the AnC estimates between the 5-Y_*LIN*_ and GEC_*AVG*_ models (*r*^2^ = 0.990; Figure 4B), as well as between the 5-Y_*LIN*_ and GEC_*LAST*_ models (*r*^2^ = 0.956; Figure 4D). This explains the relatively large typical errors and 95% limits of agreements for the AnC differences observed for the 5-Y_*LIN*_ vs. GEC_*AVG*_ and GEC_*LAST*_ models (shown in Figures 3E,F), and the small typical error and 95% limits of agreement for the 5+Y_*LIN*_ vs. GEC_*AVG*_ model (shown in Figure 3B).

Supplementary data based on two alternative polynomial models and three alternative linear models are provided in the Supplementary Tables 1, 2.

## Discussion

The main findings of the current study were that the total required MR during the 4-min TT was ∼3.9% lower when a baseline MR was included in the linear model (i.e., 5+Y_*LIN*_) compared to no inclusion of a baseline MR (i.e., 5-Y_*LIN*_) and the two GEC models (i.e., GEC_*AVG*_ and GEC_*LAST*_). The significantly higher Y-intercept in the 5+Y_*LIN*_ model resulted in a significantly lower slope of the regression line and a higher SEE. The average AnC was ∼26% lower for the 5+Y_*LIN*_ model vs. the three other models (i.e., 5-Y_*LIN*_, GEC_*AVG*_, and GEC_*LAST*_). Although the estimated anaerobic capacities for the 5-Y_*LIN*_, GEC_*AVG*_, and GEC_*LAST*_ models were very similar on a group level, the within-participant variation was relatively high, as indicated by the typical errors that ranged between 0.07 and 0.16 kJ⋅kg^–1^. The 5+Y_*LIN*_ model demonstrated smaller typical errors when compared with the GE_*AVG*_ and GE_*LAST*_ models than when compared with the 5-Y_*LIN*_ model, which was highly related to the considerably larger between-participant variation in Y-intercept values for the 5-Y_*LIN*_ model.

There are several problems associated with the various methods used to estimate the AnC (Noordhof et al., 2010, 2013). Based on previous validation data on supine one-legged dynamic knee-extensor exercise a high agreement between the indirect MAOD method and a more sophisticated direct measurement was observed (Bangsbo et al., 1990), which indicates that the MAOD method might be valid for whole-body exercise as well. When using the MAOD method several previous studies have either used a forced Y-intercept of 5 mL⋅kg^–1^⋅min^–1^, a resting $\dot{\text{V}}{\text{O}}_{2}$ measured at baseline, or an arbitrary value in the linear regression (Medbø et al., 1988; Russell et al., 2000, 2002; Bickham et al., 2002). This procedure has been suggested to increase the precision of the estimated $\dot{\text{V}}{\text{O}}_{2}$ demand (Medbø et al., 1988; Russell et al., 2000; Noordhof et al., 2011). However, the use of a Y-intercept value in the linear regression between speed (or power output) and MR (or $\dot{\text{V}}{\text{O}}_{2}$) can only be justified if it is reasonably aligned with the submaximal stages of exercise. In the current study, the inclusion of a Y-intercept value in the linear regression resulted in a significantly lower required MR, GEC, and AnC during the TT (see Figure 1). Moreover, compared to the 5-Y_*LIN*_ model, the 5+Y_*LIN*_ regression model demonstrated a significantly lower slope (3.85 vs. 4.32 W⋅kg^–1^ per m⋅s^–1^) and a higher Y-intercept (1.69 vs. 0.18 W⋅kg^–1^), which together indicate that the included Y-intercept value changed the regression equation noticeably. The SEE was considerably larger for the 5+Y_*LIN*_ than the 5-Y_*LIN*_ model, which indicates a worse fit of the regression line when a baseline MR (i.e., resting value) was added. These results suggest that the total metabolic requirement and the estimated AnC are likely to be underestimated when including a baseline MR (i.e., a modeled Y-intercept) in the linear relationship between speed and MR for treadmill running exercise. Interestingly, some previous studies have shown GEC or gross oxygen cost (i.e., VO_2_ consumed per unit of distance) to be speed independent (on a group level) (Fletcher et al., 2009; Helgerud et al., 2010; Shaw et al., 2014), indicating a zero Y-intercept for the linear regression between speed and MR (or $\dot{\text{V}}{\text{O}}_{2}$). This because GEC or gross oxygen cost would only be speed independent if the Y-intercept is zero, which is also similar for GE and the regression between power output and MR (Batliner et al., 2018; Andersson et al., 2020). Moreover, using a forced Y-intercept in the linear regression model has been observed to generate unreasonably low values of estimated AnC in running compared to cycling (Hill et al., 2002). Also, the linearity of the regression has been questioned for treadmill running (Bangsbo, 1996; Hill, 1996; Hill and Vingren, 2011). This, together with the current findings, conflicts with the traditional view that a baseline value (i.e., a Y-intercept) should be included in the linear regression when estimating the AnC during treadmill running (Medbø et al., 1988; Russell et al., 2000; Noordhof et al., 2011).

Even though it is well known that the energy equivalent per unit of VO_2_ differs considerably between fats and carbohydrates (Weir, 1949), most previous studies have used expressions of oxygen cost for determining running (or movement) economy without considering the potential influence of substrate utilization (Conley and Krahenbuhl, 1980; Daniels and Daniels, 1992; Duffield et al., 2005b; Helgerud et al., 2010). This is of particular relevance when studying heterogeneous participants or evaluating the effect of exercise intensity on running economy (Fletcher et al., 2009; Shaw et al., 2014). Due to the aforementioned factors, the current study employed two linear regression models that were based on submaximal MR, rather than submaximal $\dot{\text{V}}{\text{O}}_{2}$, to estimate the total metabolic requirement. The two linear models were also used on a second-by-second basis to estimate the instantaneous total metabolic requirement, which, compared to the traditional MAOD concept described by Medbø et al. (1988), can provide more detailed information regarding the anaerobic energy distribution during exercise. This is also an essential aspect when comparing different pacing strategies and/or repeated performances (Hettinga et al., 2006; Andersson et al., 2016).

To facilitate the comparison between the four different models, GEC during the TT was calculated from the regression equations for the 5+Y_*LIN*_ and 5-Y_*LIN*_ models and average values were similar for the 5-Y_*LIN*_, GEC_*AVG*_, and GEC_*LAST*_ models, and higher than the 5+Y_*LIN*_ model (Table 2). In the current study, GEC was observed to be speed independent on a group level, which explains the similar estimated average anaerobic capacities for the 5-Y_*LIN*_, GEC_*AVG*_, and GEC_*LAST*_ models as shown in Figure 1. However, individual data show that GEC derived from the individual 5-Y_*LIN*_ regressions, as well as the directly measured GEC, were not speed independent (see Figures 2D,E), which explains the generally high disagreement observed between the different models used for estimating the AnC (see Figure 2F). Interestingly, the inclusion of a baseline MR as a Y-intercept had a relatively large effect on the GEC values calculated from the linear regression equation (see Figure 2C vs. Figure 2E). The disagreement for GEC derived from the regression equation vs. directly measured GEC was also higher for the 5+Y_*LIN*_ than the 5-Y_*LIN*_ model as indicated by the significantly higher root mean square error. The disagreement in GEC values at the TT speed, i.e., the disagreement in GEC values computed from the 5+Y_*LIN*_ and 5-Y_*LIN*_ regressions and the GEC_*AVG*_ and GEC_*LAST*_, helps to explain the variability in the estimated anaerobic capacities between the different computational models (see Figure 2). As shown in Figure 3, the typical errors were fairly high for all the four models compared and the 5+Y_*LIN*_ generated significantly lower values of AnC in comparison to 5-Y_*LIN*_, GE_*AVG*_, and GE_*LAST*_. Inclusion of a baseline resting MR as a Y-intercept in the linear model compared to no baseline resulted in a large mean difference and typical error for the 5+Y_*LIN*_ vs. 5-Y_*LIN*_ model (Figure 3A). Based on the inconsistent individual speed vs. GEC relationships demonstrated in Figure 2E, it is likely that the use of the 5+Y_*LIN*_, GE_*AVG*_, and GE_*LAST*_ models are less reliable than the 5-Y_*LIN*_ model for estimating AnC. Therefore, to create a more robust linear relationship between speed and MR for treadmill running, it is probably wise to include some additional submaximal stages rather than adding a Y-intercept.

In a previous study, Andersson et al. (2020) demonstrated for the first time that the between-participant variation in Y-intercept values is related to the disagreement between the MAOD and GE concepts for estimating AnC. Not surprisingly, a similar finding was observed here when using GEC instead of GE, with the value of the Y-intercept of the 5-Y_*LIN*_ model being linearly related to the mean difference in anaerobic capacities between the 5-Y_*LIN*_ vs. the two GEC models (see Figures 4B,D). However, the strength of the same relationships decreased considerably for the 5+Y_*LIN*_ vs. the two GEC models (see Figures 4A,C). This was most likely due to: (1) the substantially reduced between-participant variation in the modeled Y-intercept values when including a baseline MR (i.e., a resting value); and (2) the disagreement between measured GEC and calculated GEC based on the 5+Y_*LIN*_ regression for the submaximal stages being significantly higher for the 5+Y_*LIN*_ model, as based on the higher root mean square error.

The current study is to our knowledge the first that has compared the GEC concept for estimating AnC with the more traditional MAOD method. In theory, the GEC_*LAST*_ model is very similar to the GE concept used for estimating anaerobically attributable work or AnC (Noordhof et al., 2011; Andersson and McGawley, 2018). If assuming a linear regression between power output, or speed (for running), vs. MR with a modeled Y-intercept that is similar to the resting MR, GE would increase with higher power output while an inverse relationship would be the case for the GEC of treadmill running vs. speed (Andersson and McGawley, 2018; Andersson et al., 2020). This is simply explained by the fact that the relative contribution of the baseline resting MR to the total MR decreases with higher exercise intensity (Ettema and Lorås, 2009). Due to this, both GE and GEC at supramaximal exercise intensities can be estimated from a linear regression equation between submaximal power output, or speed (for running), and MR (as shown in Figures 2A–D). Therefore, a linear regression model used for estimating AnC can also be employed as a method for estimating GEC (or GE) during high-intensity exercise. In contrast, the GEC based on the GEC_*LAST*_ or GEC_*AVG*_ models for estimating AnC can be converted to a linear regression between speed and MR where the Y-intercept would always be zero and with the slope representing the GEC_*LAST*_ or GEC_*AVG*_. For exercise using a fixed GE value for estimating AnC (Noordhof et al., 2011), it is simply the same analogy, but the linear regression between power output and MR would be best described as a slope representing the reciprocal value of GE combined with a zero Y-intercept (Andersson et al., 2020).

In the current study, the estimated anaerobic capacities were relatively low and for the four models combined, approximately one-third of the theoretical maximum proposed by Saltin (1990). However, somewhat similar values of anaerobic capacities have been observed previously for level running (Olesen, 1992; Sloniger et al., 1997). In previous literature, uphill running has also been shown to generate higher anaerobic capacities than level running, which is explained at least in part by the lower amount of activated muscle mass in the lower extremities (Olesen, 1992; Sloniger et al., 1997). It is also plausible that the degree of linearity of the relationship between submaximal speed and MR is different for running on different slopes (Olesen, 1992). For instance, Hill et al. (2002) observed unreasonably large differences in anaerobic capacities between running and cycling, with ∼39% lower values of AnC in running when employing the same MAOD method and using a forced Y-intercept of 5 mL⋅kg^–1^⋅min^–1^ as suggested by Medbø et al. (1988). Hill et al. (2002) proposed that these differences were mainly a computational effect, i.e., employing a linear regression on a relationship that was more upwardly curvilinear in running than in cycling. In the current study where running was analyzed, the inclusion of a measured and modeled (i.e., not forced) Y-intercept in the MAOD model resulted in a 25% lower value of AnC compared to no inclusion. A similar finding has been observed for uphill diagonal-stride roller-skiing (Andersson and McGawley, 2018; Andersson et al., 2020). These findings suggest that the inclusion of a Y-intercept value in the linear regression equation is likely to underestimate the AnC while running or roller-skiing (diagonal-stride) on a treadmill as it underestimates the GEC (or overestimates GE for roller-skiing) during a maximal TT. However, the inclusion of a Y-intercept might be more relevant for cycling exercise where technique is likely to be more robust over a larger range of intensities, and might thus be better aligned with a measured Y-intercept than running or roller-skiing. For instance, Figure 2E shows large individual variability in GEC vs. speed, which indicates that the effect of speed on running biomechanics and GEC is highly individual, and adding a measured Y-intercept may change the speed-GEC relationship considerably as shown in Figure 2.

In a previous study by Hill and Vingren (2011), the relationship between speed and MR was found to be upwardly curvilinear for running but linear for cycle ergometry. In order to make a thorough evaluation of the linearity of the data in the current study, the results generated by two alternative polynomial models and three alternative linear models were compared with the conventional 5-Y_*LIN*_ and GEC_*LAST*_ models, and these data are presented as Supplementary Tables. The results in Supplementary Table 1 reveal that the AnC estimates were not significantly different for the five alternative models when compared to the conventional 5-Y_*LIN*_ and GEC_*LAST*_ models, indicating that the submaximal relationship between speed and submaximal MR for the five submaximal stages can be considered linear. The results in Supplementary Table 2 also reveal that the agreements between the two polynomial models and the other models were relatively poor. Interestingly, the comparison between the AnC estimates for the 5-Y_*POL*_ vs. 5-Y_*LIN*_ models resulted in a high typical error (0.20 kJ⋅kg^–1^). This indicates that polynomial models can be problematic to use on data that are mainly linear due to the issue of so-called overfitting, which has also been observed previously for diagonal-stride roller-skiing (Andersson et al., 2020).

In the current study, a mixed group of female and male recreational, endurance-trained athletes was recruited. It is possible that between-participant variability in physiological characteristics could have an impact on the agreement between the different models that were used to estimate AnC, and that such variability could be higher for a mixed-sex athlete group. For example, it is possible that between-participant variation in a physiological variable such as $\dot{\text{V}}{\text{O}}_{2max}$ could influence the agreement between the GEC_*LAST*_ and 5-Y_*LIN*_ models of estimating AnC. This is because the relative influence of the baseline (i.e., resting) MR on GEC becomes smaller with increasing exercise intensity, and $\dot{\text{V}}{\text{O}}_{2max}$ is directly related to the participant’s highest absolute submaximal exercise intensity. However, the range in $\dot{\text{V}}{\text{O}}_{2max}$ was relatively small in the current study and as a result, it is unlikely that sex differences *per se* would have influenced the results to a major extent.

## Perspectives and Conclusion

This study aimed to compare four different models of estimating AnC during high-intensity treadmill running and to methodologically examine the agreement between the four models. Two of the four models were based on a linear relationship between submaximal speed and MR (i.e., 5+Y_*LIN*_ and 5-Y_*LIN*_) and two were based on a fixed GEC value (i.e., GEC_*AVG*_ and GEC_*LAST*_). The GEC_*AVG*_ and GEC_*LAST*_ models were introduced because of the similarities to the GE concept that has been used for estimating AnC/work capacity during cycling (Serresse et al., 1988; Noordhof et al., 2011).

The main findings of the current study were that GEC was found to be speed independent on a group level and that 5-Y_*LIN*_, GEC_*AVG*_, and GEC_*LAST*_ generated similar values of AnC, while the 5+Y_*LIN*_ model generated ∼26% lower values of AnC. The lower anaerobic capacities estimated with the 5+Y_*LIN*_ could be related to the significantly lower slope of the regression line and the higher Y-intercept value, which resulted in a lower GEC value (based on the regression equation) during the 4-min TT effort. Although the 5-Y_*LIN*_, GEC_*AVG*_, and GEC_*LAST*_ generated similar values of AnC, they should not be used interchangeably due to the profound individual variability, as indicated by the large typical errors and the large SEM. The 5-Y_*LIN*_ model might be the most reliable and valid model out of the four models that were studied and as such, this model is recommended for estimating AnC during treadmill running exercise. This is because GEC was not observed to be speed independent on an individual basis and GEC calculated from the 5-Y_*LIN*_ linear regression equation was more similar to the directly measured GEC at the five submaximal intensities.

## Data Availability Statement

The raw data supporting the conclusions of this article will be made available by the authors, without undue reservation.

## Ethics Statement

The studies involving human participants were reviewed and approved by The Regional Ethical Review Board of Umeå University, Umeå, Sweden. The patients/participants provided their written informed consent to participate in this study.

## Author Contributions

EA and KM designed the study. KM, EA, and GB were responsible for the data collection, drafted the final manuscript, approved the final version to be published, and agreed to be accountable for all aspects of the work. EA analyzed and interpreted the data, and wrote the first draft of the manuscript. All authors contributed to the article and approved the submitted version.

## Conflict of Interest

The authors declare that the research was conducted in the absence of any commercial or financial relationships that could be construed as a potential conflict of interest.

## Publisher’s Note

All claims expressed in this article are solely those of the authors and do not necessarily represent those of their affiliated organizations, or those of the publisher, the editors and the reviewers. Any product that may be evaluated in this article, or claim that may be made by its manufacturer, is not guaranteed or endorsed by the publisher.
