# Supplementary material for: Anaerobic Capacity in Running: The Effect of Computational Method
Source: Front Physiol. 2021 Aug 4;12:708172. doi: 10.3389/fphys.2021.708172 (PMC8371633; doi:10.3389/fphys.2021.708172)
Supplement: Supplementary file 1 [file Data_Sheet_1.docx]

Supplementary Tables

**Table S1**. Mean ± SD Y-intercept, linear coefficient (L_coeff_), quadratic coefficient (Q_coeff_), and standard error of estimate (SEE) for two polynomial models and four linear models of estimating anaerobic capacity, as well as average gross energy cost (GEC_TT_avg_), average metabolic requirement (MR_TT_req_), anaerobic capacity (AnCap), and accumulated oxygen deficit (ΣO_2_ deficit) during the 4-min time trial (TT) for the six aforementioned models and the gross energy cost procedure based on the last submaximal stage (GEC_LAST_).

|  | 5+Y_POL_ | 5-Y_POL_ | 5-Y_LIN_ | 4-Y_LIN_ | 3-Y_LIN_ | 2-Y_LIN_ | GEC_LAST_ | F-value | *P*-value | ES | SEM |
| --- | --- | --- | --- | --- | --- | --- | --- | --- | --- | --- | --- |
| Y-intercept | 1.79 | 2.49 | 0.18 | -0.05 | -0.32 | -0.28 | - | F_1.43,20_ | *P* = 0.047 | η^2^ = 0.222 | - |
| (W∙kg^-1^) | ± 0.23*^b-f^* | ± 4.52 | ± 1.71 | ± 2.01 | ± 1.93 | ± 2.68 |  | = 4.0 |  |  |  |
| L_coeff_ | 3.29 | 2.81 | 4.32 | 4.39 | 4.47 | 4.46 | - | F_1.28,18_ | *P* = 0.043 | η^2^ = 0.238 | - |
| (W∙kg^-1^ / m∙s^-1^) | ± 0.62*^c-f^* | ± 2.89 | ± 0.56 | ± 0.63 | ± 0.62 | ± 0.83 |  | = 4.4 |  |  |  |
| Q_coeff_ | 0.17 | 0.24 | - | - | - | - | - | F_1.00,14_ | *P* = 0.519 | η^2^ = 0.030 | - |
| (W∙kg^-1^ / m∙s^-2^) | ± 0.17 | ± 0.47 |  |  |  |  |  | = 0.4 |  |  |  |
| SEE | 0.12 | 0.12 | 0.13 | 0.11 | 0.08 | - | - | F_2.57,36_ | *P* = 0.026 | η^2^ = 0.208 | 0.05 |
| (W∙kg^-1^) | ± 0.04 | ± 0.06 | ± 0.05 | ± 0.07 | ± 0.07 |  |  | = 3.7 |  |  |  |
| GEC_TT_avg_ | 4.47 | 4.52 | 4.37 | 4.39 | 4.41 | 4.41 | 4.38 | F_2.07,29_ | *P* = 0.067 | η^2^ = 0.174 | 0.14 |
| (J∙kg^-1^∙m^-1^) | ± 0.36 | ± 0.43 | ± 0.30 | ± 0.31 | ± 0.32 | ± 0.35 | ± 0.27 | = 2.9 |  |  |  |
| MR_TT_req_ | 104 | 105 | 102 | 102 | 103 | 103 | 102 | F_2.05,29_ | *P* = 0.061 | η^2^ = 0.180 | 3.44 |
| (% of MR_ae_peak_) | ± 7 | ± 10 | ± 5 | ± 5 | ± 5 | ± 6 | ± 4 | = 3.1 |  |  |  |
| AnCap | 0.84 | 0.90 | 0.72 | 0.75 | 0.76 | 0.77 | 0.74 | F_2.01,28_ | *P* = 0.069 | η^2^ = 0.174 | 0.18 |
| (kJ∙kg^-1^) | ± 0.30 | ± 0.44 | ± 0.20 | ± 0.23 | ± 0.24 | ± 0.28 | ± 0.15 | = 2.9 |  |  |  |
| ΣO_2_ deficit | 40 | 43 | 35 | 36 | 37 | 37 | 35 | F_2.01,28_ | *P* = 0.069 | η^2^ = 0.174 | 8.41 |
| (mL∙kg^-1^) | ± 14 | ± 21 | ± 9 | ± 11 | ± 11 | ± 13 | ± 7 | = 2.9 |  |  |  |

Abbreviations: 5+Y_POL_ and 5-Y_POL_, the 5 × 4-min second-degree polynomial models with the baseline metabolic rate as a modeled Y-intercept either included (5+Y) or excluded (5-Y); 5-Y_LIN_, the 5 × 4-min linear models without using a baseline metabolic rate as a modeled Y-intercept; 4-Y_LIN_, the 4 × 4-min linear model (i.e., same as 5-Y_LIN_ but excluding the first submaximal stage); 3-Y_LIN_, the 3 × 4-min linear model (i.e., same as 5-Y_LIN_ but excluding the first two submaximal stages); 2-Y_LIN_, the 2 × 4-min linear model (i.e., same as 5-Y_LIN_ but excluding the first three submaximal stages); L_coeff_, linear coefficient; Q_coeff_, quadratic coefficient; ES, effect size; SEM, standard error of measurement; GEC_TT_avg_, average GEC during the TT; MR_TT_req_, required metabolic rate during the TT; MR_ae_peak_, peak aerobic metabolic rate during the TT.

F-values, *P*-values, and eta squared effect size (η^2^) were obtained by a one-way ANOVA. *^b-f^*Significantly different from 5-Y_POL_, 5-Y_LIN_, 4-Y_LIN_, 3-Y_LIN_, and 2-Y_LIN_, all *P* < 0.05. *^c-f^*Significantly different from 5-Y_LIN_, 4-Y_LIN_, 3-Y_LIN_, and 2-Y_LIN_, all *P* < 0.05.

**Table S2**. Mean difference (MEAN_diff_) between seven different models of estimating anaerobic capacity from a 4-minute self-paced running time-trial.

|  | MEAN_diff_  (kJ∙kg^-1^) | LOA  (kJ∙kg^-1^) | TE | ES*_hg_av_* |
| --- | --- | --- | --- | --- |
| 5+Y_POL_ vs 5-Y_POL_ | -0.06 | -0.62 to 0.50 | 0.20 | -0.1 |
| 5+Y_POL_ vs 5-Y_LIN_ | 0.11* | -0.13 to 0.36 | 0.09 | 0.4 |
| 5+Y_POL_ vs 4-Y_LIN_ | 0.09* | -0.11 to 0.29 | 0.07 | 0.3 |
| 5+Y_POL_ vs 3-Y_LIN_ | 0.07 | -0.25 to 0.40 | 0.12 | 0.3 |
| 5+Y_POL_ vs 2-Y_LIN_ | 0.06 | -0.20 to 0.33 | 0.10 | 0.2 |
| 5+Y_POL_ vs GEC_LAST_ | 0.10 | -0.37 to 0.57 | 0.17 | 0.4 |
| 5-Y_POL_ vs 5-Y_LIN_ | 0.17 | -0.45 to 0.80 | 0.23 | 0.5 |
| 5-Y_POL_ vs 4-Y_LIN_ | 0.15 | -0.39 to 0.68 | 0.19 | 0.4 |
| 5-Y_POL_ vs 3-Y_LIN_ | 0.13 | -0.36 to 0.63 | 0.18 | 0.4 |
| 5-Y_POL_ vs 2-Y_LIN_ | 0.12 | -0.39 to 0.64 | 0.19 | 0.3 |
| 5-Y_POL_ vs GEC_LAST_ | 0.16 | -0.55 to 0.87 | 0.26 | 0.5 |
| 5-Y_LIN_ vs 4-Y_LIN_ | -0.02 | -0.13 to 0.08 | 0.04 | -0.1 |
| 5-Y_LIN_ vs 3-Y_LIN_ | -0.04 | -0.24 to 0.16 | 0.07 | -0.2 |
| 5-Y_LIN_ vs 2-Y_LIN_ | -0.05 | -0.31 to 0.21 | 0.09 | -0.2 |
| 5-Y_LIN_ vs GEC_LAST_ | -0.01 | -0.25 to 0.22 | 0.09 | -0.1 |
| 4-Y_LIN_ vs 3-Y_LIN_ | -0.02 | -0.21 to 0.17 | 0.07 | -0.1 |
| 4-Y_LIN_ vs 2-Y_LIN_ | -0.03 | -0.26 to 0.21 | 0.09 | -0.1 |
| 4-Y_LIN_ vs GEC_LAST_ | 0.01 | -0.29 to 0.31 | 0.11 | 0.0 |
| 3-Y_LIN_ vs 2-Y_LIN_ | -0.01 | -0.28 to 0.26 | 0.10 | 0.0 |
| 3-Y_LIN_ vs GEC_LAST_ | 0.03 | -0.25 to 0.30 | 0.10 | 0.1 |
| 2-Y_LIN_ vs GEC_LAST_ | 0.04 | -0.35 to 0.42 | 0.14 | 0.2 |

Abbreviations: LOA, limits of agreement; TE, typical error; ES*_hg_av_*, Hedges’s *g_av_* effect size; 5+Y_POL_ and 5-Y_POL_, the 5 × 4-min second-degree polynomial models with the baseline metabolic rate as a modeled Y-intercept either included (5+Y) or excluded (5-Y); 5-Y_LIN_, the 5 × 4-min linear models without using a baseline metabolic rate as a modeled Y-intercept; 4-Y_LIN_, the 4 × 4-min linear model (i.e., same as 5-Y_LIN_ but excluding the first submaximal stage); 3-Y_LIN_, the 3 × 4-min linear model (i.e., same as 5-Y_LIN_ but excluding the first two submaximal stages); 2-Y_LIN_, the 2 × 4-min linear model (i.e., same as 5-Y_LIN_ but excluding the first three submaximal stages); GEC_LAST_, the gross energy cost procedure based on the last submaximal stage. *P*-values for MEAN_diff_ were obtained by a paired-sample *t*-test. **P* < 0.01.
